# Analysis of microorganisms isolated from tracheal aspirate cultures and their antibiotic susceptibility profiles: a retrospective study from 2018 to 2022

**DOI:** 10.3389/fmed.2026.1770208

**Published:** 2026-02-10

**Authors:** Erkan Sanmak, İsmail Davarci, Gül Şahika Gökdemir, Gökhan Güler, Hayri Canbaz, Zeynep Ayaydin, Mehmet Kabak, Barış Çil

**Affiliations:** 1 Ministry of Health, Head of the Department of Health Professions, Ankara, Türkiye; 2 Department of Medical Microbiology, Faculty of Medicine, Trakya University, Edirne, Türkiye; 3 Department of Physiology, Faculty of Medicine, Mardin Artuklu University, Mardin, Türkiye; 4 Ministry of Health, General Directorate of Health Services, Ankara, Türkiye; 5 Department of Emergency Medicine, Yenimahalle Training and Research Hospital, Yıldırım Beyazıt University, Ankara, Türkiye; 6 Department of Medical Microbiology, Faculty of Medicine, Mardin Artuklu University, Mardin, Türkiye; 7 Department of Chest Diseases, Faculty of Medicine, Mardin Artuklu University, Mardin, Türkiye; 8 Department of Chest Diseases, Mardin Training and Research Hospital, Mardin, Türkiye

**Keywords:** antimicrobial resistance, gram-negative bacteria, intensive care unit, multidrug-resistant organisms, tracheal aspirate, ventilator-associated pneumonia

## Abstract

**Background:**

To determine the distribution of microorganisms isolated from tracheal aspirate (TA) cultures and their antimicrobial susceptibility patterns, and to assess resistance differences between intensive care unit (ICU) – and ward-derived isolates as well as temporal trends across years.

**Methods:**

Tracheal aspirate specimens obtained at a tertiary-care center between 2018 and 2022 were retrospectively reviewed. Only growth meeting laboratory acceptance criteria for causative pathogens was analyzed (semi-quantitative culture thresholds with cytologic quality control). Bacterial identification was performed using automated systems, and antimicrobial susceptibility testing was interpreted according to EUCAST standards. In addition to descriptive analyses, annual resistance trends and a joinpoint regression analysis (annual percent change) were conducted.

**Results:**

Of all causative isolates, 83.8% were Gram-negative. The most frequent pathogens were *Klebsiella* spp., *Acinetobacter* spp., and *Pseudomonas* spp. For *Klebsiella* spp., resistance to cephalosporins and fluoroquinolones was generally >90%, meropenem >80%, whereas imipenem showed comparatively higher susceptibility. In *Acinetobacter* spp., resistance was very high to most agents, with amikacin showing the lowest resistance. In *Pseudomonas* spp., resistance rates ranged from 40% to 55%, and amikacin emerged as the most active agent. Resistance was systematically higher in ICU-derived isolates than in ward isolates. Joinpoint analysis identified a single breakpoint around 2020; resistance trajectories during 2018–2020 were heterogeneous, with increases observed for some organism–antimicrobial combinations, followed by divergent patterns thereafter.

**Conclusion:**

The predominance of Gram-negative pathogens and the high resistance burden in our center support locally tailored Gram-negative coverage for empiric therapy alongside early de-escalation. Temporal patterns underscore the need to update empiric policies using annual local surveillance data and to reinforce infection control and antimicrobial stewardship, particularly in ICUs.

## Introduction

Pneumonia is an acute inflammatory consolidation of lung parenchyma caused by infectious agents, including bacteria, viruses, fungi, and parasites. Bacterial pneumonia refers to inflammation involving one or more lobes due to bacterial infection. Hospital-acquired pneumonia (HAP) is defined as pneumonia developing ≥48 h after hospital admission; when associated with mechanical ventilation, it is termed ventilator-associated pneumonia (VAP) (1).

According to the Global Burden of Disease Study 2023, lower respiratory infections were responsible for approximately 2.50 million deaths worldwide in 2023 across all age groups, remaining the leading infectious cause of death globally and ranking among the top causes of mortality overall (2).

Antibiotic resistance, driven by inappropriate and excessive antimicrobial use, has become an escalating global health threat (3). Pneumonia caused by multidrug-resistant Gram-negative bacteria (MDR-GNB) is increasingly prevalent and adversely affects patient outcomes. This trend is characterized–particularly in hospital settings–by a shift toward Gram-negative etiologies and their rapid dissemination (4–6).

The aim of the present study was to delineate the spectrum of microorganisms isolated from tracheal aspirate (TA) cultures, to characterize their antimicrobial resistance profiles according to EUCAST criteria, and to analyze temporal trends, thereby providing evidence to inform empiric therapy and antimicrobial stewardship strategies.

## Materials and methods

The microbiological procedures and analytical approaches applied to evaluate pathogen distribution, antimicrobial resistance, and temporal trends over the study period are described below.

### Study design

This study was designed as a retrospective, observational, single-center, laboratory-based study conducted at Mardin Training and Research Hospital, the largest hospital in Mardin province located in southeastern Türkiye near the Syrian border. The hospital is a 400-bed tertiary-care institution serving as a regional referral center for critically ill patients. Tracheal aspirate (TA) cultures submitted to the Medical Microbiology Laboratory between January 2018 and December 2022 were reviewed. Patient confidentiality was maintained throughout, and no personally identifiable information was used in the analyses.

### Specimen collection, incubation, and evaluation

Tracheal aspirate samples were obtained from patients who required suctioning as part of routine clinical care. During sample collection, a sterile specimen trap was connected to the ventilator circuit, and tracheal secretions were collected during routine suctioning. In patients with highly viscous secretions, normal saline was administered to facilitate effective suctioning. Samples were transported promptly to the laboratory under conditions preserving pathogen viability and inoculated semi-quantitatively onto 5% sheep blood agar, chocolate agar, and Eosin Methylene Blue agar. Plates were incubated aerobically at 37 °C.

All culture results meeting laboratory acceptability criteria–defined as <10 epithelial cells per low-power field–were reviewed individually. Semi-quantitative cultures were graded as rare, 1+, 2+, 3+, or 4+ based on the number of plate quadrants demonstrating growth. Only isolates showing 4+ growth or 3+ pure growth were included in the analyses. The following were reported as normal oral flora in respiratory specimens and not analyzed as pathogens: viridans streptococci and bacteria from the genera *Gemella*, *Granulicatella*, *Abiotrophia*, *Peptostreptococcus*, *Veillonella*, *Prevotella*, *Porphyromonas*, *Rothia*, *Lactobacillus*, and *Bacillus*. Only the first isolate per patient was included; subsequent isolates from the same patient were excluded. Isolates were included only when microbiologically confirmed as causative agents of infection.

### Antimicrobial susceptibility testing

Antimicrobial susceptibility testing (AST) was performed using conventional methods and an automated system (VITEK-2, bioMérieux, Marcy-l’Étoile, France). The antimicrobial panel was selected in accordance with recommendations of the Turkish Society of Microbiology (7). Susceptibility interpretation followed EUCAST (The European Committee on Antimicrobial Susceptibility Testing) criteria applicable to 2018–2022 (8). For analysis, isolates categorized as intermediate were treated as susceptible.

### Data collection and statistical analysis

Laboratory records were abstracted into a structured dataset including patient demographics, clinical unit, specimen type, organism identification, and AST profiles. Given the retrospective observational design, analyses were primarily descriptive, with additional time-trend analyses performed to evaluate changes in antimicrobial resistance over the study period. Descriptive statistics were calculated first: continuous variables are reported as mean ± standard deviation (SD) and median (min–max), and categorical variables as frequency (n) and percentage (%).

To examine whether antimicrobial resistance patterns changed over time, temporal trends were assessed using joinpoint regression to identify statistically significant changes over time, potential breakpoints (joinpoints), and Annual Percent Change (APC) for each segment. The Average Annual Percent Change (AAPC) was also computed for the entire study period. Ninety-five percent confidence intervals were reported, and statistical significance was set at *p* < 0.05.

### Ethical approval

This retrospective study was approved by the Mardin Artuklu University Non-Interventional Clinical Research Ethics Committee (Decision No. 2025/1-13). Patient confidentiality was maintained throughout, and no personally identifiable information was used in the analyses.

## Results

### Patient and specimen characteristics

A total of 1,610 TA culture-positive specimens collected between 2018 and 2022 were included in the analysis. The majority of specimens originated from intensive care units (91.4%), with level 3 ICUs accounting for more than 80% of ICU-derived samples (Table 1). The mean age of the patients was 55.6 ± 27.5 years (median 59; IQR 38–78; range 0–99). In Türkiye, intensive care units are classified into three levels according to patients’ clinical status and organ support requirements, in accordance with the Regulation on the Procedures and Principles of Intensive Care Services in Inpatient Health Facilities (9). Level 1 ICUs provide care for patients requiring close clinical monitoring without advanced organ support, level 2 ICUs serve patients requiring limited organ support (typically single-organ dysfunction), and level 3 ICUs represent the highest level of care, designated for patients with severe clinical conditions requiring advanced life-support interventions. Outside the ICU setting, specimens were most frequently submitted from the Internal Medicine department (*n* = 86), followed by Chest Diseases (*n* = 36). Smaller numbers were received from Otorhinolaryngology (*n* = 6), Thoracic Surgery (*n* = 5), and Neurology (*n* = 3), while Pediatrics and Plastic Surgery each contributed one specimen (*n* = 1).

**TABLE 1:** Distribution of tracheal aspirate culture-positive specimens by sex, year, and care setting/ICU characteristics.

Sex	*n*	%
Female	628	39.0
Male	982	61.0
Total	1610	100
**Year**		
2018	211	13.1
2019	272	16.9
2020	399	24.8
2021	378	23.5
2022	350	21.7
Total	1610	100.0
**Care setting (ward/ICU)**		
Ward	138	8.6
ICU	1472	91.4
Total	1610	100.0
**ICU type**		
COVID ICU	114	7.7
Pediatric ICU	218	14.8
General ICU	1130	76.8
Coronary Care Unit (CCU)	10	0.7
Total	1472	100.0
**ICU level**		
Level 1 ICU	2	0.1
Level 2 ICU	286	19.4
Level 3 ICU	1184	80.5
Total	1472	100.0

ICU, intensive care unit.

### Distribution of isolated microorganisms

Of all causative isolates, 83.8% were Gram-negative, 15.2% were Gram-positive, and 1.0% were yeasts. The most frequently isolated microorganisms were *Klebsiella* spp., *Acinetobacter* spp., and *Pseudomonas* spp. Overall, 91.4% of isolates were recovered from intensive care unit (ICU) patients (Table 2).

**TABLE 2:** Distribution of microorganisms isolated from tracheal aspirate cultures across intensive care unit subtypes and wards (2018–2022).

Microorganism	General ICU		Pediatric ICU		COVID ICU		Coronary Care Unit (CCU)		Ward		Total	
	*n*	%	*n*	%	*n*	%	*n*	%	*n*	%	*n*	%
*Klebsiella* spp.	347	30.7	37	17.0	35	30.7	1	10.0	28	20.3	448	27.8
*Acinetobacter* spp.	297	26.3	46	21.1	50	43.9	1	10.0	12	8.7	406	25.2
*Pseudomonas* spp.	130	11.5	71	32.6	4	3.5	1	10.0	39	28.3	245	15.2
*E. coli*	66	5.8	15	6.9	2	1.8	1	10.0	9	6.5	93	5.8
*S. aureus*	60	5.3	14	6.4	3	2.6	4	40.0	10	7.2	91	5.7
*Corynebacterium* spp.	54	4.8	3	1.4	1	0.9	1	10.0	0	0.0	59	3.7
*Serratia* spp.	36	3.2	10	4.6	1	0.9	0	0.0	4	2.9	51	3.2
CoNs	32	2.8	6	2.8	3	2.6	1	10.0	4	2.9	46	2.9
*S. maltophilia*	6	0.5	7	3.2	8	7.0	0	0.0	12	8.7	33	2.0
*S. pneumoniae*	17	1.5	2	0.9	0	0.0	0	0.0	4	2.9	23	1.4
*Proteus* spp.	13	1.2	3	1.4	0	0.0	0	0.0	6	4.3	22	1.4
*Enterococcus* spp.	12	1.1	0	0.0	3	2.6	0	0.0	1	0.7	16	1.0
*Enterobacter* spp.	10	0.9	0	0.0	0	0.0	0	0.0	2	1.4	12	0.7
*Morganella* spp.	8	0.7	0	0.0	1	0.9	0	0.0	0	0.0	9	0.6
*Candida* spp.	7	0.6	0	0.0	0	0.0	0	0.0	2	1.4	9	0.6
*Candida albicans*	6	0.5	0	0.0	1	0.9	0	0.0	0	0.0	7	0.4
Other	29	2.6	4	1.8	2	1.8	0	0.0	5	3.6	40	2.5
Total	1130	100.0	218	100.0	114	100.0	10	100.0	138	100.0	1610	100.0

### Antimicrobial resistance patterns: ICU vs. ward

Among the three most frequently isolated pathogens, the lowest resistance in *Klebsiella* spp. was observed for imipenem (36.2%), whereas for *Acinetobacter* spp. and *Pseudomonas* spp., the lowest resistance rates were recorded for amikacin (76.3% and 21.9%, respectively). Antimicrobial resistance rates were consistently higher among ICU isolates compared with ward isolates for most agents, particularly carbapenems and aminoglycosides, as summarized in Table 3. This pattern underscores the substantial antimicrobial pressure in intensive care settings and has important implications for empiric treatment strategies in critically ill patients.

**TABLE 3:** Antimicrobial resistance rates (%) of the three most frequently isolated microorganisms from tracheal aspirate cultures, stratified by care setting (ICU vs. ward), 2018–2022.

Antimicrobials	*Klebsiella* spp.				*Acinetobacter* spp.				*Pseudomonas* spp.			
	Ward		ICU		Ward		ICU		Ward		ICU	
	*n*	%	*n*	%	*n*	%	*n*	%	*n*	%	*n*	%
Ampicillin	13	100.0	131	100.0	–	–	–	–	–	–	–	–
Cefazolin	8	100.0	104	96.2	–	–	–	–	–	–	–	–
Pip/Tazo	26	76.9	366	91.3	–	–	–	–	37	43.2	154	53.2
Cefuroxime	12	100.0	131	93.9	–	–	–	–	–	–	–	–
Ceftriaxone	9	88.9	99	92.9	–	–	–	–	–	–	–	–
Ceftazidime	26	80.8	366	95.4	–	–	–	–	37	43.2	155	52.3
Amikacin	6	33.3	44	72.7	1	0.0	37	78.4	5	20.0	27	22.2
Ciprofloxacin	21	81.0	337	90.5	10	100.0	292	98.6	32	43.8	139	44.6
Levofloxacin	16	62.5	276	91.3	11	90.9	267	98.1	32	56.3	141	50.4
TMP/SMX	1	0.0	15	86.7	0	–	22	95.5	–	–	–	–
Imipenem	22	27.3	312	36.9	11	90.9	286	97.6	35	40.0	149	53.0
Meropenem	19	47.4	271	81.2	7	100.0	209	98.6	29	48.3	120	46.7
Cefepime	23	73.9	352	94.6	–	–			35	37.1	147	48.3
Gentamicin	20	55.0	329	81.8	10	90.0	288	92.4	–	–	–	–

ICU, intensive care unit; Pip/Tazo, piperacillin–tazobactam; TMP/SMX, trimethoprim–sulfamethoxazole.

### Temporal trends in antimicrobial resistance

Temporal trend analysis was performed to evaluate changes in antimicrobial resistance patterns over the study period. Resistance trends demonstrated heterogeneous patterns during 2018–2020, with increases observed for several organism–antimicrobial combinations, while others showed stable or declining trajectories.

**FIGURE 1:**
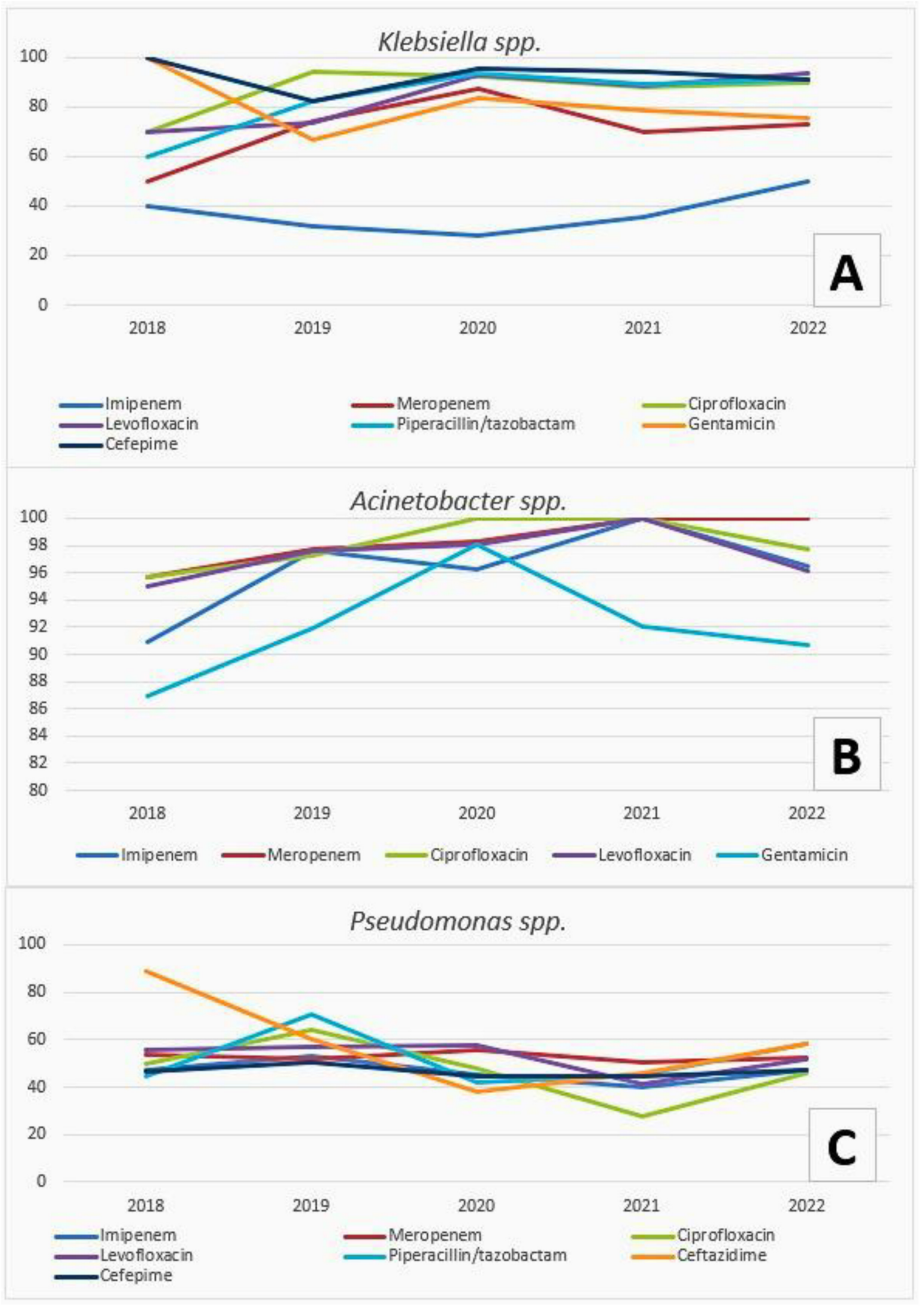
Yearly trends in antimicrobial resistance rates (%) for the three most frequently isolated bacteria from tracheal aspirate cultures obtained from hospitalized patients, predominantly from intensive care units, between 2018 and 2022. **(A)** *Klebsiella* spp., **(B)** *Acinetobacter* spp., and **(C)** *Pseudomonas* spp.

Figure 1 presents annual antimicrobial resistance trends for selected clinically relevant agents against the three most frequently isolated pathogens. The trends reflect overall isolate data from hospitalized patients, predominantly derived from intensive care units. The antimicrobials shown were selected to represent major drug classes commonly used in the empiric treatment of pneumonia and were limited to agents with sufficient annual testing data across the study period. Overall, resistance patterns varied by pathogen and antimicrobial agent, with no uniform directional trend observed across all combinations.

**FIGURE 2:**
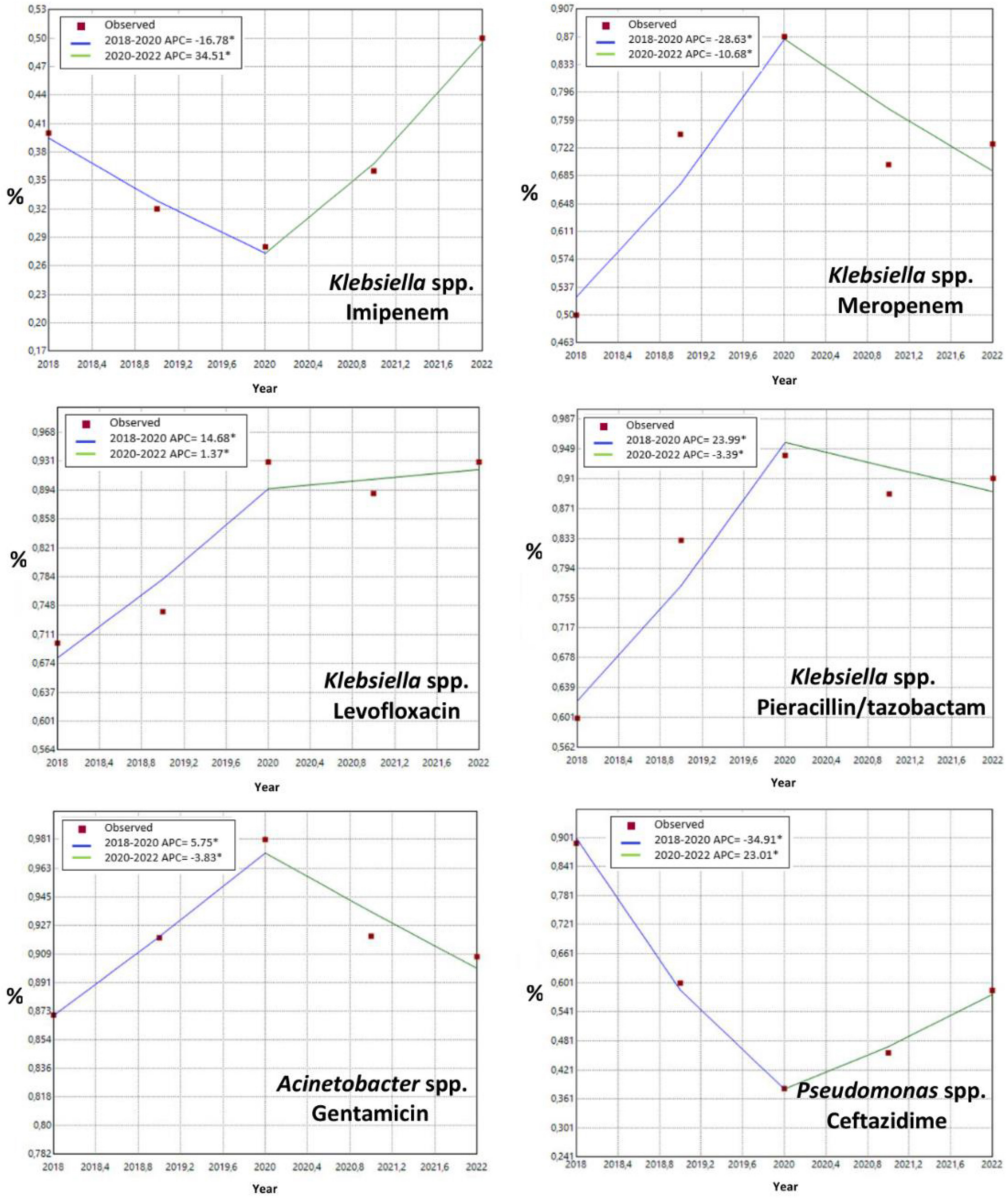
Joinpoint curves depicting changes in resistance rates over time for various antimicrobials against *Klebsiella* spp., *Acinetobacter* spp., and *Pseudomonas* spp. Joinpoint regression was used to identify statistically significant changes in resistance trends over time, expressed as annual percent change (APC) and potential breakpoints. Only organism–antimicrobial combinations with statistically significant joinpoints are shown. *Indicates that the Annual Percent Change (APC) is significantly different from zero at the alpha = 0.05 level.

Joinpoint regression (annual percent change, APC) identified a breakpoint around 2020 across several organism–antimicrobial combinations. Resistance trajectories during 2018–2020 were heterogeneous, with increasing trends observed for some combinations, while others demonstrated stable or declining patterns, followed by divergent post-2020 trajectories depending on the pathogen and antimicrobial agent (Figure 2). Although joinpoint analysis was conducted for all organism–antimicrobial combinations, only those exhibiting statistically significant breakpoints were included in Figure 2. Detailed joinpoint regression results, including annual percent change (APC), average annual percent change (AAPC), 95% confidence intervals, and *p*-values for all organism–antimicrobial combinations, are provided in Supplementary Table 1.

## Discussion

This study provides a comprehensive analysis of the microbiological characteristics of TA specimens from patients with suspected pneumonia. It delineates antimicrobial resistance (AMR) distributions, examines their relationships with demographic subgroups and contributing clinical units, and situates the findings within national and global AMR trends to inform contemporary debate and decisions on empiric therapy/antibiotic stewardship. In addition, the analysis of temporal trends offers a longitudinal perspective on the evolution of antimicrobial resistance in this setting.

Globally, HAP ranks second among nosocomial infections and occurs in approximately 0.5%–1.7% of hospitalized patients. It remains a leading cause of death attributable to healthcare-associated infections, with incidence ranging from about 5 to >20 cases per 1,000 admissions (10, 11).

Prior studies show that HAP is most often associated with Gram-negative bacteria (GNB) such as *Klebsiella pneumoniae*, *Acinetobacter baumannii*, *Pseudomonas aeruginosa*, and *Escherichia coli* (1, 12, 13). In VAP, reported GNB prevalence ranges from 76.1% to 88.3%, with highly multidrug-resistant (MDR) *P. aeruginosa* and *A. baumannii* frequently predominating (14–16). In a large cohort, HAP and VAP among ICU patients were associated with 30-days mortality risk increases of 82% and 38%, respectively (17), and GNB account for roughly 50%–80% of HAP/VAP etiologies (18). Among ICU populations in the United States and Europe, proportions of HAP and VAP due to GNB have been reported at 61.5% and 76.1% (19); higher proportions were observed in Egyptian pediatric cohorts (91.67% and 87.8%) (20) and in Iran (72.2% and 84.6%) (21). In our series, the proportion of GNB isolated from TA was 83.8%, aligning with the upper bounds of the literature and supporting prioritization of GNB coverage in empiric regimens. At the same time, TA cultures may reflect colonization; therefore, early antibiotic de-escalation guided by culture/susceptibility results and the routine surveillance of local resistance trends, coupled with strengthened infection control and stewardship, should be prioritized.

Multidrug-resistant, extensively drug-resistant (XDR), and pan-drug-resistant (PDR) organisms–particularly GNB–are increasingly isolated in HAP/VAP and have been linked to mortality rates exceeding 50% (22). In our cohort, *Klebsiella* spp., *Acinetobacter* spp., and *Pseudomonas* spp. were most frequently isolated. Resistance profiles showed >90% resistance to cephalosporins and fluoroquinolones and >80% to meropenem among *Klebsiella* spp., with comparatively higher susceptibility to imipenem. In *Acinetobacter* spp., resistance was generally >90% to most agents, with the lowest resistance to amikacin. In *Pseudomonas* spp., resistance to most agents was in the ∼50% range, again with the lowest resistance to amikacin. This pattern suggests limited clinical utility of third-generation cephalosporins and fluoroquinolones for empiric therapy in our setting. The combination of high meropenem resistance and comparatively lower imipenem resistance may reflect a composite effect of local carbapenemase repertoires and porin/efflux alterations; thus, carbapenems should be used selectively, with early de-escalation and susceptibility-guided individualization. Empiric approaches should favor anti-pseudomonal β-lactams with robust Gram-negative coverage and combination therapy where appropriate, followed by prompt narrowing once culture/AST results become available. Concurrently, reinforcing infection control and antimicrobial stewardship, maintaining up-to-date local antibiograms, and investigating potential clonal dissemination via molecular typing may help reduce resistance burden and adverse clinical outcomes.

The predominance of samples from intensive care units likely reflects greater exposure to invasive devices, prolonged ventilation, and repeated broad-spectrum antibiotic use. Our findings therefore support prioritizing GNB coverage in ICU empiric regimens, enforcing early de-escalation based on culture/AST, and strengthening infection prevention measures such as environmental cleaning and resistance surveillance. The temporal trend analysis provides additional insight into the dynamics of antimicrobial resistance over the study period. Joinpoint regression identified a breakpoint around 2020, with heterogeneous resistance trajectories observed before and after this time point. This temporal shift plausibly coincides with the COVID-19 pandemic period and may reflect multiple, interrelated factors. During the pandemic, intensive care units experienced substantial changes in patient case mix, including increased numbers of critically ill patients requiring prolonged mechanical ventilation and invasive supportive therapies. Empirical and often broad-spectrum antibiotic use increased globally, particularly during the early phases of the pandemic, driven by concerns regarding bacterial co-infections and diagnostic uncertainty. In parallel, healthcare systems faced significant strain, which may have temporarily compromised routine infection prevention and antimicrobial stewardship activities. Changes in hospital admission patterns, antimicrobial availability, and laboratory workflows during this period may also have contributed to the observed heterogeneity in post-2020 resistance trajectories. Although causal relationships cannot be established in this retrospective analysis, these pandemic-related disruptions provide a plausible context for the breakpoint identified in the joinpoint analysis.

From a clinical perspective, the high prevalence of multidrug-resistant Gram-negative bacteria and the consistently elevated resistance rates observed among ICU isolates underscore the need for locally tailored empirical antibiotic strategies for pneumonia, particularly in critical care settings. Our findings support the regular updating of institutional empirical treatment guidelines based on contemporary local resistance data and ICU-specific antibiograms. Given the absence of a single agent achieving optimal susceptibility thresholds, initial combination therapy may be warranted in selected high-risk patients, followed by prompt de-escalation guided by culture and susceptibility results. These data further highlight the importance of sustained antimicrobial stewardship interventions aimed at optimizing empirical therapy, minimizing unnecessary broad-spectrum antibiotic use, and reducing resistance selection pressure.

Although pneumonia can be recognized on clinical grounds, etiologic identification requires laboratory-capable specimens (23). In practice, sputum quality can be limited by contamination with normal respiratory flora; obtaining sputum is often difficult in children and older adults; and transthoracic lung aspiration, while generally safe, has limited feasibility (24). Routine culture, organism identification, and AST require different specimen types and pre-analytical handling, have modest diagnostic yield, and may take 48–72 h to report. TA obtained with a clean suction catheter is a non-invasive, low-cost, low-risk method for procuring lower respiratory tract specimens–especially in recently intubated patients (25). However, the propensity of the endotracheal tube and central airways toward colonization complicates distinction of invasive infection from colonization; clinical scoring systems and quantitative culture thresholds are therefore recommended (25–27). Adding TA to routine diagnostics can significantly increase microbiological yield in pneumonia cases that would otherwise remain “culture negative” (25).

Antimicrobial selection should be guided by the percent susceptible (%S) threshold and clinical severity. Current WHO guidance recommends agents with %S ≥ 90 for empiric treatment of severe infections (28). In our setting, no single agent reliably meets this threshold for *Klebsiella*, *Acinetobacter*, or *Pseudomonas* spp.; consequently, initial combination therapy is often warranted, with prompt de-escalation upon receipt of culture/AST data.

Gram-negative bacteria pneumonia can be acquired via aspiration of bacteria from the upper aerodigestive tract, inhalation of aerosols, or hematogenous spread from distant foci (e.g., urinary tract, gastrointestinal tract) into the alveoli; among these, aspiration is a common mechanism for both HAP and community-acquired pneumonia (29, 30). MDR-GNB may be acquired in both community and hospital settings. Reported risk factors include prolonged hospitalization, prior MDR-GNB colonization or infection, high local resistance prevalence, ICU admission, mechanical ventilation, and surgical (31–33). They are also more frequent in older adults, in patients with prior antibiotic exposure, chronic lung diseases (e.g., COPD, bronchiectasis), diabetes mellitus, immunosuppressive states (e.g., HIV, malignancy), prior hospitalization, and chronic alcoholism (1).

At the community level–particularly in low-income settings–unregulated access to inexpensive antibiotics without prescription promotes inadequate pathogen eradication, treatment failure, and selection of resistant GNB, thereby increasing the prevalence of drug-resistant organisms (34). Additional drivers include suboptimal infection control and stewardship, limited vaccine coverage targeting GNB, high in-hospital GNB burden with downstream dissemination via hospital effluents, acquisition of resistance through mobile genetic elements (e.g., plasmids, transposons), increasing comorbidity burden, population aging, and high virulence determinants (35–37). These forces foster selection, persistence, and spread of MDR/XDR/PDR organisms, leading to antibiotic treatment failure, increased mortality and morbidity, and substantial rises in the costs of treatment and prevention (38).

Joinpoint analysis indicated a shared breakpoint around 2020, with a shift from generally increasing resistance during 2018–2020 to heterogeneous post-2020 trajectories (stabilization/decrease in some organism–agent pairs and partial rebound in others). This pattern is plausibly consistent with pandemic-era changes in patient mix (ICU load, ventilation duration), shifts in antibiotic utilization (treatment preferences, drug availability), and fluctuations in infection control/stewardship practices; evolving EUCAST breakpoints and laboratory workflow differences across years may also have contributed. Joinpoint statistics do not imply causality and may be influenced by unmeasured confounders. Even so, the post-2020 divergence supports annual local surveillance–informed updates of empiric policies, persistent application of early de-escalation, and reinforcement of ICU-focused infection prevention measures.

This study has several limitations that should be acknowledged. First, its single-center, retrospective design, based on microbiology laboratory records, may limit the generalizability of the findings to other institutions with different patient populations, antimicrobial prescribing patterns, and infection control practices. Second, the analysis relied exclusively on TA specimens. Although TA sampling is widely used in mechanically ventilated patients because of its feasibility and low procedural risk, it may reflect airway colonization rather than true invasive infection, and the absence of comparative respiratory specimens (e.g., bronchoalveolar lavage fluid or sputum) limits direct cross-specimen comparisons. Third, the predominance of intensive care unit specimens may restrict extrapolation of the results to non-ICU wards or less severely ill patient populations. In addition, pandemic-era changes during 2020–2021, including shifts in patient case mix and antimicrobial use, may have influenced microbiological distributions and resistance patterns. Finally, molecular resistance mechanisms (e.g., ESBL or carbapenemase genes) were not investigated; therefore, phenotypic resistance profiles could not be genotypically confirmed. These limitations highlight the need for prospective, multicenter studies integrating clinical, radiologic, microbiological, and molecular data.

In conclusion, the predominance of GNB and the high resistance burden in our center necessitate carefully planned empiric Gram-negative coverage, early de-escalation, regular antibiogram updates, and robust infection control and stewardship programs.

## Data Availability

The raw data supporting the conclusions of this article will be made available by the authors, without undue reservation.
