# Supplementary material for: Analysis of microorganisms isolated from tracheal aspirate cultures and their antibiotic susceptibility profiles: a retrospective study from 2018 to 2022
Source: Front Med (Lausanne). 2026 Feb 10;13:1770208. doi: 10.3389/fmed.2026.1770208 (PMC12929516; doi:10.3389/fmed.2026.1770208)
Supplement: Supplementary file 1 [file Table_1.docx]

**Supplementary Table 1**. Annual percent change (APC) and joinpoint analysis of antimicrobial resistance trends in tracheal aspirate isolates, 2018–2022

| **Pathogen** | **Antimicrobial** | **Time Interval (Years)** | **APC (%)** | **95% CI (Lower / Upper)** | ***p*-value** | **Joinpoint Year** | **AAPC (%)** |
| --- | --- | --- | --- | --- | --- | --- | --- |
| *Klebsiella* spp. | Imipenem | 2018-2020 | -16.779 | -19.9809 / -13.3049 | <0.000001 | 2020 | 5.8028 |
| *Klebsiella* spp. | Imipenem | 2020-2022 | 34.5126 | 29.0252 / 39.9558 | <0.000001 |  |  |
| *Klebsiella* spp. | Meropenem | 2018-2020 | 28.6261 | 8.3495 / 54.0313 | 0.000400 | 2020 | 7.1853 |
| *Klebsiella* spp. | Meropenem | 2020-2022 | -10.6814 | -256780 / 602153 | 0.198760 |  |  |
| *Klebsiella* spp. | Ciprofloxacin | 2018-2022 | 3.3425 | -12.8714 / 22.2089 | 0.601480 | None | 3.3425 |
| *Klebsiella* spp. | Levofloxacin | 2018-2020 | 14.6813 | 5.1085 / 25.5948 | <0.000001 | 2020 | 7.8184 |
| *Klebsiella* spp. | Levofloxacin | 2020-2022 | 1.3662 | -7.5874 / 10.6242 | 0.711458 |  |  |
| *Klebsiella* spp. | Pip/Tazo | 2018-2020 | 23.9896 | 13.3853 / 36.5061 | <0.000001 | 2020 | 9.4484 |
| *Klebsiella* spp. | Pip/Tazo | 2020-2022 | -3.3875 | -12.4784 / 6.0707 | 0.444311 |  |  |
| *Klebsiella* spp. | Gentamicin | 2018-2022 | -3.7683 | -13.2518 / 6.4995 | 0.457109 | None | -3.7683 |
| *Klebsiella* spp. | Cefepime | 2018-2022 | -0.6396 | -5.3902 / 4.3396 | 0.747451 | None | -0.6396 |
| *Acinetobacter* spp. | Imipenem | 2018-2022 | 1.4280 | -3.3665 / 6.4075 | 0.466307 | None | 1.4280 |
| *Acinetobacter* spp. | Meropenem | 2018-2022 | 1.1179 | 0.3513 / 1.8898 | 0.002400 | None | 1.1179 |
| *Acinetobacter* spp. | Ciprofloxacin | 2018-2022 | -74.2427 | -94.2636 / 11.0907 | 0.068386 | None | -74.2427 |
| *Acinetobacter* spp. | Levofloxacin | 2018-2022 | 0.4743 | -1.8243 / 2.7408 | 0.630274 | None | 0.4743 |
| *Acinetobacter* spp. | Gentamicin | 2018-2020 | 5.7499 | 2.8531 / 8.8751 | <0.000001 | 2020 | 0.8474 |
| *Acinetobacter* spp. | Gentamicin | 2020-2022 | -3.8278 | -6.5594 / -1.1251 | 0.001600 |  |  |
| *Pseudomonas* spp. | Imipenem | 2018-2022 | -2.8131 | -17.7406 / 14.4614 | 0.591082 | None | -2.8131 |
| *Pseudomonas* spp. | Meropenem | 2018-2022 | 0.9464 | -5.7193 / 3.7756 | 0.543491 | None | 0.9464 |
| *Pseudomonas* spp. | Ciprofloxacin | 2018-2022 | -9.5937 | -30.0245 / 15.9489 | 0.410718 | None | -9.5937 |
| *Pseudomonas* spp. | Levofloxacin | 2018-2022 | -4.6825 | -12.9644 / 4.1647 | 0.307139 | None | -4.6825 |
| *Pseudomonas* spp. | Pip/Tazo | 2018-2022 | 1.0718 | -16.7493 / 21.6754 | 0.924215 | None | 1.0718 |
| *Pseudomonas* spp. | Ceftazidime | 2018-2020 | -34.9066 | -37.8855 / -31.6330 | <0.000001 | 2020 | -10.5187 |
| *Pseudomonas* spp. | Ceftazidime | 2020-2022 | 23.0063 | 16.9913 / 28.9614 | <0.000001 |  |  |
| *Pseudomonas* spp. | Cefepime | 2018-2022 | -9.876 | -5.7301 / 3.7432 | 0.657469 | None | -9.876 |

Pip/Tazo : Piperacillin/tazobactam, CI: Confidence Interval
